# c-Met, CREB1 and EGFR are involved in miR-493-5p inhibition of EMT via AKT/GSK-3β/Snail signaling in prostate cancer

**DOI:** 10.18632/oncotarget.19398

**Published:** 2017-07-19

**Authors:** Song Wang, Xiao Wang, Jiangfeng Li, Shuai Meng, Zhen Liang, Xin Xu, Yi Zhu, Shiqi Li, Jian Wu, Mingjie Xu, Alin Ji, Yiwei Lin, Ben Liu, Xiangyi Zheng, Bo Xie, Liping Xie

**Affiliations:** ^1^ Department of Urology, The First Affiliated Hospital, School of Medicine, Zhejiang University, Hangzhou, Zhejiang Province, People’s Republic of China; ^2^ Department of Urology, Zhejiang Provincial People’s Hospital, Hangzhou, Zhejiang Province, People’s Republic of China; ^3^ Department of Urology, Tongde Hospital of Zhejiang Province, Hangzhou, Zhejiang Province, People’s Republic of China

**Keywords:** prostate cancer, miR-493-5p, c-Met, CREB1, EGFR

## Abstract

miR-493-5p downregulation has emerged as a critical player in cancer progression yet, the underlying mechanisms of miR-493-5p expression pattern and its function in prostate cancer remains to be elucidated. Here, we illustrate that miR-493-5p is frequently downregulated in prostate cancer, at least partially due to altered DNA methylation. miR-493-5p functions as a tumor suppressor in prostate cancer cells. c-Met, CREB1 and EGFR are downstream target genes of miR-493-5p. miR-493-5p inhibits EMT via AKT/GSK-3β/Snail signaling in prostate cancer. Taken together, our study identified c-Met, CREB1, EGFR and miR-493-5p establish a regulatory loop in prostate cancer, which could prove useful in the development of effective and therapies against prostate cancer.

## INTRODUCTION

Prostate cancer is accepted as the most commonly diagnosed cancer among men worldwide, and ranks as the second cause of cancer-specific death in the United States [1]. Therefore, it is important to understand its carcinogenic mechanisms of prostate cancer and develop novel therapeutic targets.

MicroRNAs (miRNAs) are known as small non-coding RNAs (20∼23 nucleotides) that comprise a new class of target gene mediators, acting by accelerating the degradation and/or blocking the translation of their target mRNAs [2, 3]. In our previous studies, we identified a series of miRNAs involved in prostate cancer proliferation, migration and invasion, including miR-449a, miR-195-5p, and miR-330 [4-6]. However, the biological function of miRNAs in prostate cancer is still unclear.

Based on the studies about expression profiles of miRNA in prostate cancer, we focused on the expression pattern and function of miR-493-5p. miR-493-5p locates at 14q32.2, which is an important miRNA in DLK1-DIO3 region. Previous studies indicated that miR-493-5p was relevant to the carcinogenesis and progression of many types of cancers, including colon cancer, gastric cancer, and bladder cancer [7]. Nevertheless, the function of miR-493-5p in prostate cancer has not been well elucidated.

Here, we explored the function of miR-493-5p in suppression of prostate cancer proliferation and migration. Furthermore, we identified c-Met, CREB1, EGFR and miR-493-5p establish a regulatory loop in prostate cancer.

## RESULTS

### miR-493-5p is downregulated in prostate cancer

**Figure 1:**
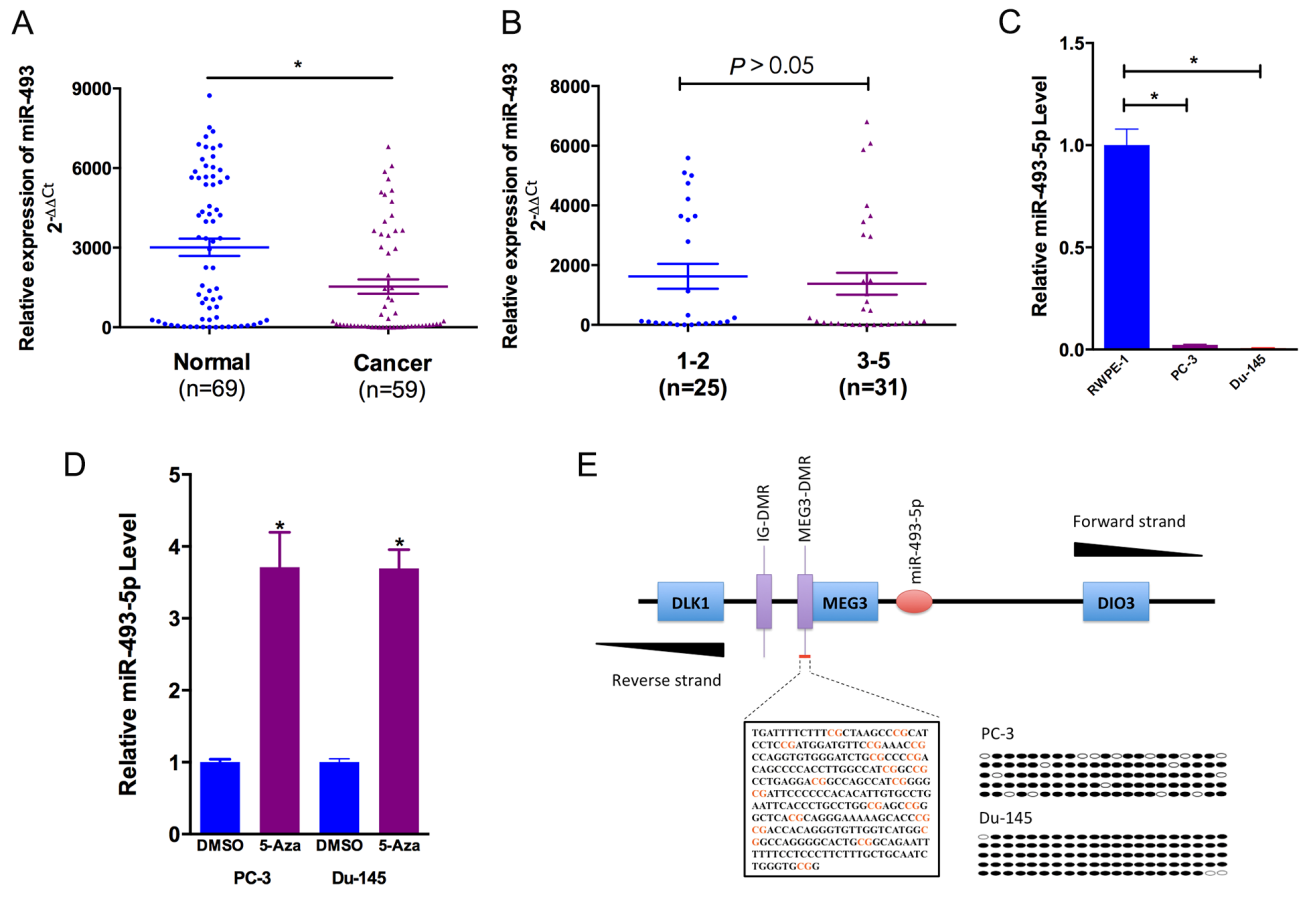
miR-493-5p is frequently downregulated in prostate cancer and is regulated by DNA methylation **(A)** Statistical analysis indicated that miR-493-5p expression was significantly lower in prostate cancer blood samples than in benign prostate hyperplasia blood samples. **(B)** No statistical difference was observed between miR-493-5p expression in high-grade prostate cancer (GGG 3-5) and low-grade prostate cancer. **(C)** miR-493-5p expression was significantly downregulated in castrated resistant prostate cancer cell lines (PC-3 and Du-145) compared to RWPE-1. **(D)** The demethylating agent 5-aza-dC stimulated miR-493-5p expression compared to DMSO-treated samples. **(E)** The regions analyzed by BSP are indicated. The open and filled circles represent the unmethylated and methylated CpG islands, respectively. Five clones from each cell line were analyzed. Error bars represent the S.E. obtained from three independent experiments; *P<0.05.

PCR analysis demonstrated that the expression of miR-493-5p was lower in prostate cancer blood samples (the patient’s whole blood) compared to benign prostate hyperplasia blood samples (the patient’s whole blood) (*P* < 0.05, Figure 1A). We further compared miR-493-5p expression in high-grade prostate cancer (GGG 3-5) and low-grade prostate cancer (GGG 1-2) [8]. However, no significant difference was observed (*P* > 0.05, Figure 1B). Clinical characteristics of the prostate cancer patients were listed in Table 1. To further evaluate miR-493-5p expression in prostate cancer, we performed quantitative real-time PCR (qRT-PCR) in RWPE-1, PC-3 and Du-145 cell lines. Compared to RWPE-1 (the human prostate epithelial cell line), the expression of miR-493-5p was downregulated in PC-3 and Du-145 (the castrated resistant prostate cancer (CRPC) cell lines) (Figure 1C). The above results were consistent with previously published data, indicating that miR-493-5p played an important role in inhibiting prostate cancer progression.

**Table 1:** Clinical characteristics of the prostate cancer patients

	No.	Age	PSA (ng/ml)	Prostate volumn (ml)
BPH group	69	63.6±7.8	9.8±7.4	43.4±26.7
Prostate cancer group	59	70.5±6.0	52.7±104.3	42.9±24.2

DNA methylation played an important role in microRNA regulation expression [9]. 5-aza-2′-deoxycytidine (short for 5-Aza), a DNA methyltransferase inhibitor, significantly increased miR-493-5p expression in PC-3 and Du-145 (Figure 1D). The CpG Island Searcher Program (http://www.urogene.org/methprimer) was searched to find CpG islands upstream of the miR-493-5p. The identified CpG island was evaluated by sodium bisulfite sequencing assay, and 84.8% and 97.1% of CpG islands were methylated in PC-3 and Du-145, which were dramatically increasing (Figure 1E). These data further verified the involvement of methylation in miR-493-5p silencing.

### miR-493-5p overexpression inhibits cell proliferation, cell motility and EMT

**Figure 2:**
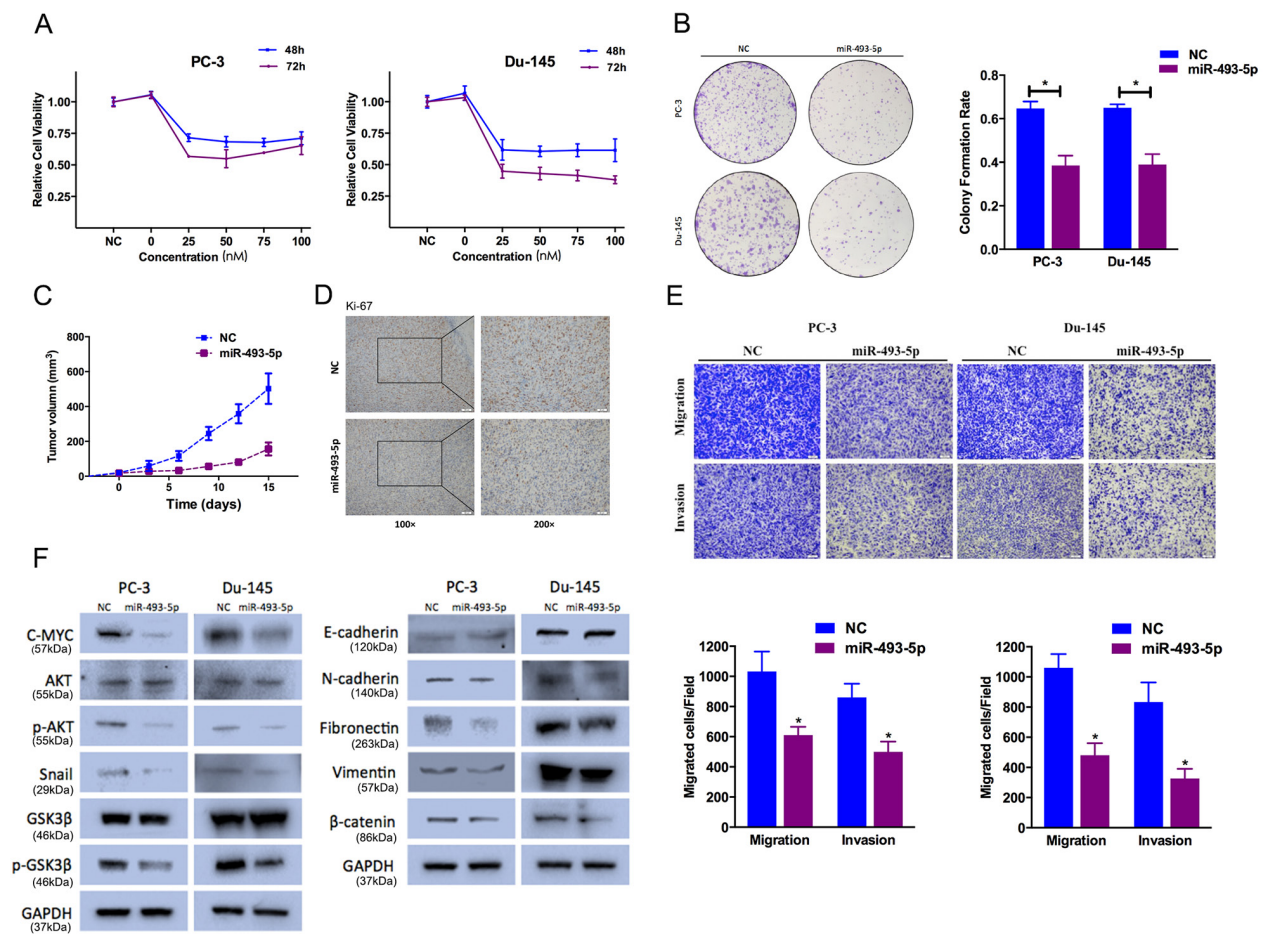
Effect of miR-493-5p on prostate cancer cell proliferation and cell motility **(A)** CCK-8 assay. The relative cell viability of the miR-493-5p-treated groups of PC-3 and Du-145 cells was lower than that of NC-treated groups (cell viability of 0 nM was regarded as 1.0). **(B)** Colony-formation assay (representative wells are presented). The colony-formation rate was lower for miR-493-5p (50 nM)-transfected groups compared to NC (50 nM)-transfected groups. **(C, D)** Tumor xenograft model. Tumor growth curves indicated that tumors in the miR-493-5p group grew more slowly. Decreased Ki-67 expression was also detected in miR-493-5p-treated tumors. **(E)** Transwell assay (representative micrographs are presented). miR-493-5p (50 nM) impaired the motility of PC-3 and Du-145 cells. **(F)** Western blot analysis. miR-493-5p (50 nM) inhibited EMT and AKT/GSK-3β signaling-related protein expression in PC-3 and Du-145 cells. Error bars represent the S.E. obtained from three independent experiments; *P<0.05. Scale bar = 100 μm.

We transfected PC-3 and Du-145 with miR-493-5p mimics, and investigated its function on cell proliferation. miR-493-5p overexpression significantly decreased proliferation in PC-3 and Du-145 by CCK-8 and colony formation assays (Figure 2A, 2B). Besides, we found inhibition of miR-493-5p expression partially rescues miR-493-5p-induced suppression of cell proliferation (Supplementary Figure 1A). We examined the proliferation rates of PC-3 in the absence or presence of miR-493-5p overexpression after its s.c. implantation into BALB/c mice. miR-493-5p significantly inhibited tumor growth *in vivo* (Figure 2C). We also confirmed that tumor derived from miR-493-5p-treated cells expressed higher level of miR-493-5p than the tumors from NC-treated group (data not shown). IHC staining analysis indicated that compared to tumors from the NC-treated group, tumors derived from miR-493-5p-treated group illustrated lower Ki-67 level (Figure 2D). These data demonstrated that miR-493-5p inhibited prostate cancer cell growth.

We found that miR-493-5p significantly suppressed PC-3 and Du-145 cell motility ability in Transewell assays (Figure 2E). Besides, we found inhibition of miR-493-5p expression partially rescues miR-493-5p-induced suppression of cell motility (Supplementary Figure 1B). EMT is a critical event in cancer progression. We examined the expression of EMT protein markers in our study. miR-493-5p overexpression upregulated the expression of E-cadherin (epithelial marker), and downregulated the expression of N-cadherin, Fibronectin, Vimentin (mesenchymal markers), and Snail (EMT-related transcription factors). We found similar results by immunofluorescence staining analysis (Supplementary Figure 2). Besides, we found inhibition of miR-493-5p expression partially rescues miR-493-5p-induced suppression of EMT (Supplementary Figure 1C). We next investigated the potential mechanisms for miR-493-5p-inhibited EMT. Previous study indicated that activation of phosphorylated AKT increased Snail nuclear localization and transcriptional activating function, thus inducing cell migration and EMT. Upon treatment with miR-493-5p, phosphorylated AKT (Ser473) expression was significantly decreasing in prostate cancer cells. Besides, phosphorylated GSK-3β (Ser9) was also decreasing in miR-493-5p-treated group (Figure 2F). These data showed that miR-493-5p suppressed EMT in prostate cancer cells via inhibiting AKT/GSK-3β/Snail pathway.

### c-Met, EGFR and CREB1 are downstream targets of miR-493-5p

**Figure 3:**
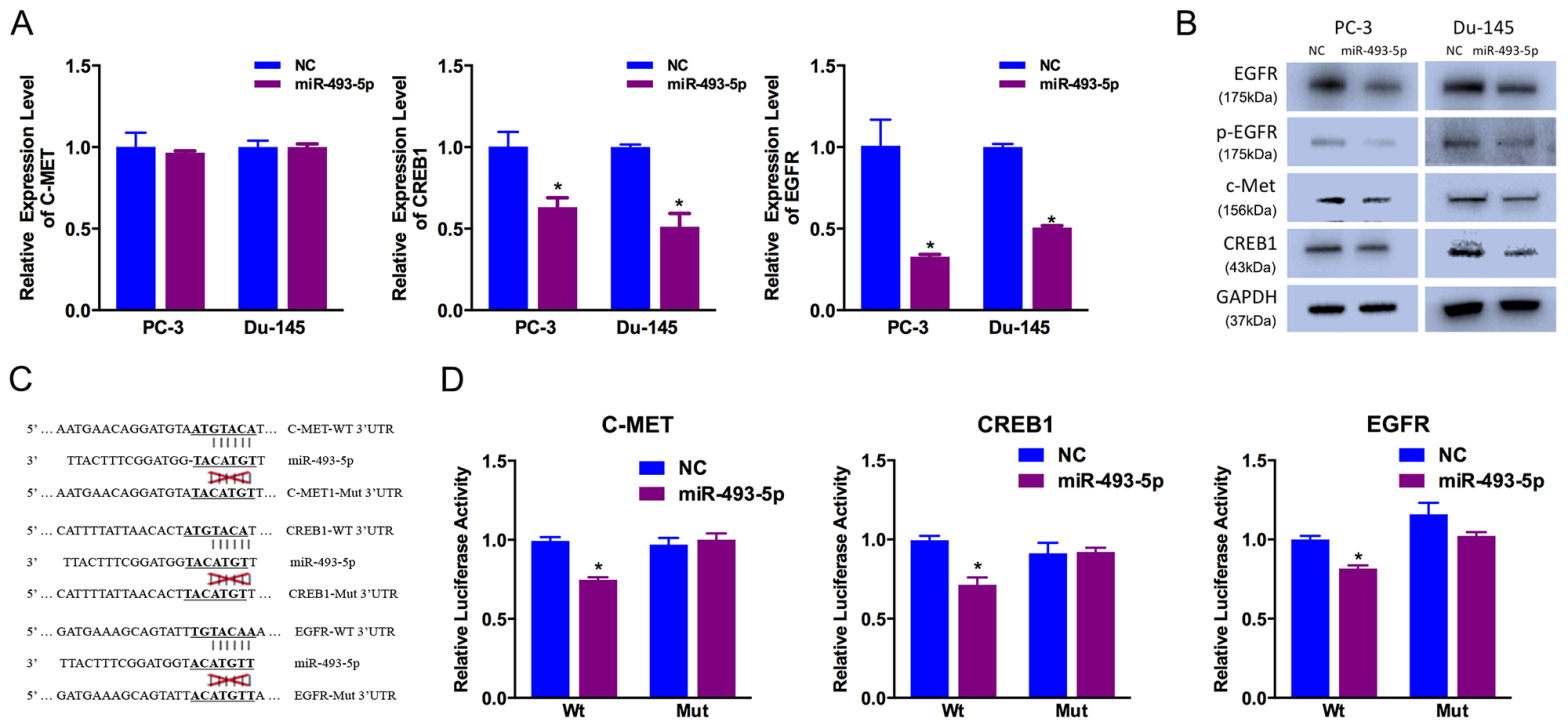
c-Met, CREB1 and EGFR are direct miR-493-5p targets **(A)** Decreased CREB1 and EGFR expression was observed in miR-493-5p-transfected PC-3 and Du-145 cells via qRT-PCR. **(B)** Decreased c-Met, CREB1 and EGFR expression was observed in miR-493-5p-transfected PC-3 and Du-145 cells via western blot. **(C)** The miR-493-5p-targeting sites in c-Met, CREB1 and EGFR 3′-UTRs were mutated. **(D)** miR-493-5p significantly suppressed the luciferase activity of vector that carried the c-Met, CREB1 and EGFR 3′-UTRs but not control vector; Error bars represent the S.E. obtained from three independent experiments; *P<0.05.

To identify potential downstream targets of miR-493-5p, we used miRanda and TargetScan online databases for analysis. Considering the candidate target genes predicted by the two online databases and the function of miR-493-5p, we chose c-Met, CREB1 and EGFR as candidate targets. Previous studies have shown that c-Met, CREB1 and EGFR are key cell motility and proliferation regulators, respectively [9]. We examined decreased expression of CREB1 and EGFR in miR-493-5p-treated prostate cancer cells at both mRNA and protein level (Figure 3A, 3B). Besides, decreased expression of c-Met was found at protein level, not mRNA level, which indicated miR-493-5p inhibited the translation of c-Met, not degradated the mRNA of c-Met (Figure 3A, 3B).

To further identify the relationship between miR-493-5p and potential target genes, luciferase reporter assays were conducted to determine whether miR-493-5p directly interacted with the 3′-UTRs (untranslated region) of EGFR, c-Met and CREB1 (Figure 3C). miR-493-5p overexpression drastically decreased the level of relative luciferase activity of the 3 targeted genes, and the level of relative luciferase activity of the control vectors (mutated vectors) was not affected by miR-493-5p overexpression (Figure 3D). These results indicated that miR-493-5p directly regulates the expression of c-Met, CREB1 and EGFR

### c-Met silencing suppresses prostate cancer cell motility and EMT

**Figure 4:**
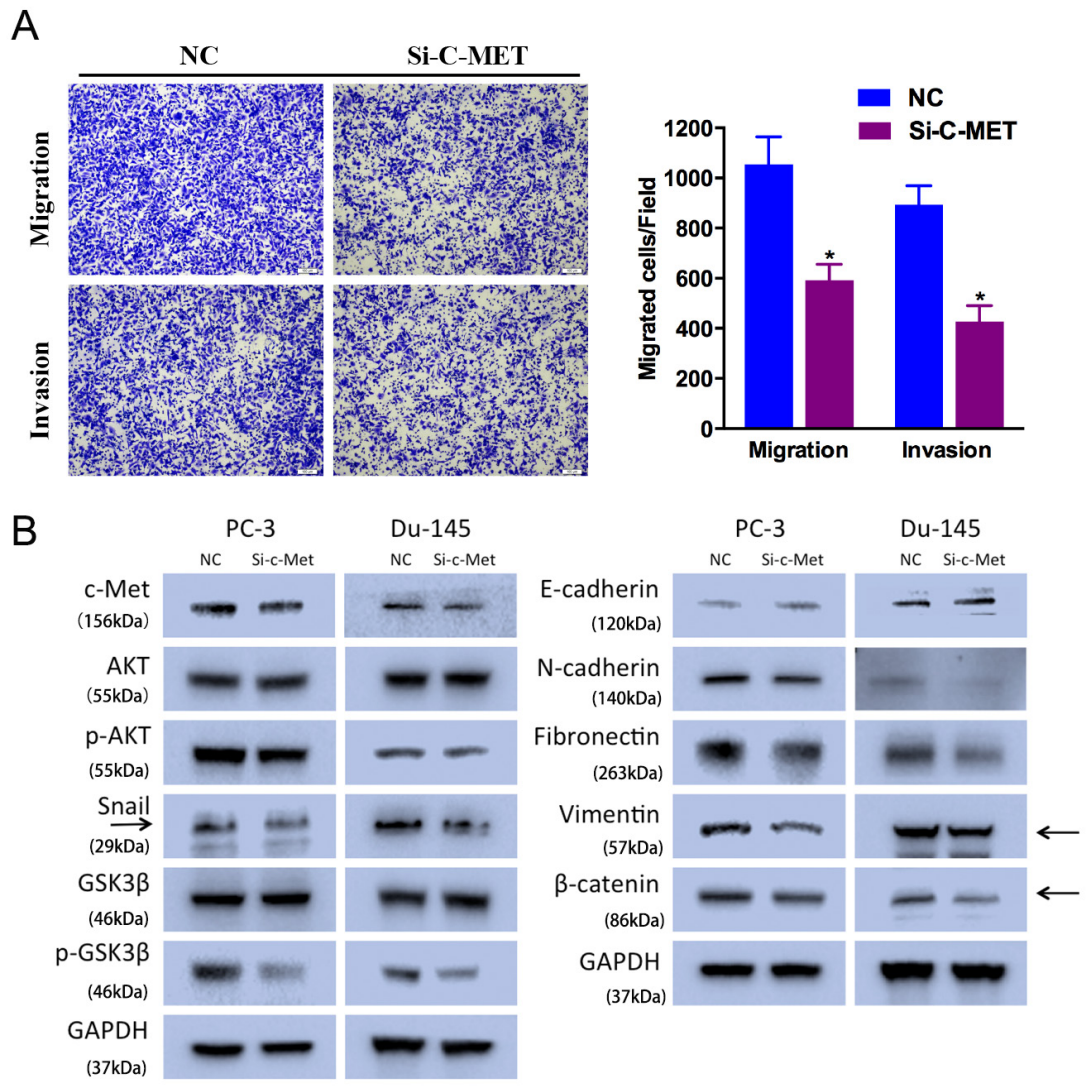
c-Met knockdown suppresses prostate cancer cell motility **(A)** Transwell assay (representative micrographs are presented). Si-c-Met (50 nM) impaired PC-3 cell motility. **(B)** Western blot analysis. Si-c-Met (50 nM) inhibited EMT-related protein expression in PC-3 and Du-145 cells. Error bars represent the S.E. obtained from three independent experiments; *P<0.05. Scale bar = 100 μm.

To avoid off-target phenomena, we use three different and effective siRNAs in our studies. The following results were obtained from one of the three siRNAs as a representative. We performed Transewell assays to identify the function of c-Met in prostate cancer cell motility. We found that Si-c-Met significantly suppressed PC-3 cell migration and invasion ability (Figure 4A).

As expected, c-Met downregulation suppressed AKT/GSK-3β/Snail signaling pathway. Si-c-Met-transfected cells exhibited lower level of N-cadherin, Fibronectin, Vimentin, Snail, phosphorylated AKT (Ser473), and phosphorylated GSK-3β (Ser9), and higher level of E-cadherin (Figure 4B). And c-Met expression was also included as a control showing the silenced target.

### CREB1 silencing suppresses prostate cancer cell proliferation, motility and EMT

**Figure 5:**
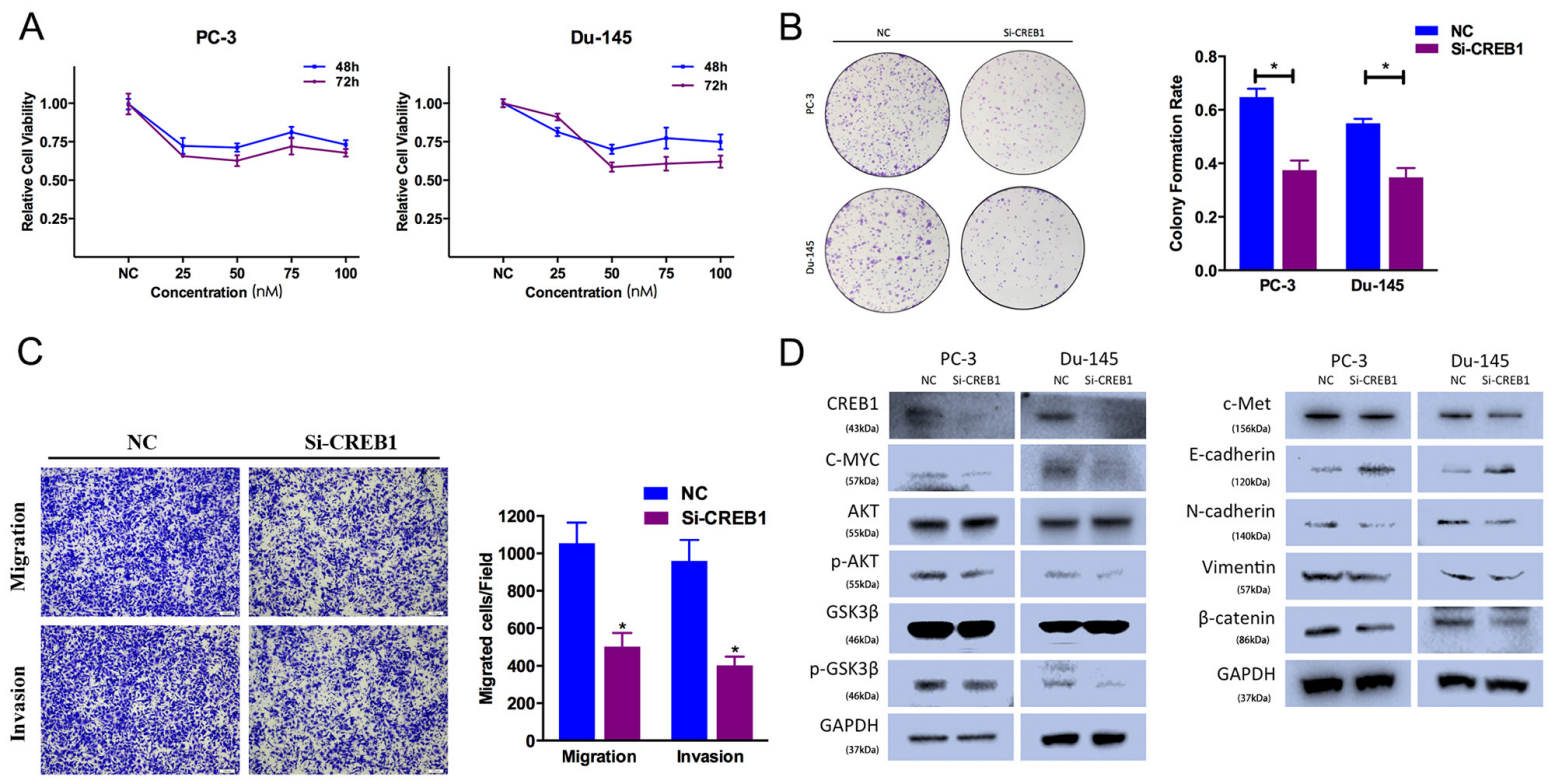
CREB1 knockdown suppresses prostate cancer cell proliferation and motility **(A)** CCK-8 assay. The relative cell viability of the Si-CREB1-treated groups of PC-3 and Du-145 cells was lower than that of NC-treated groups (cell viability of 0 nM was regarded as 1.0). **(B)** Colony-formation assay (representative wells are presented). The colony-formation rate was lower for Si-CREB1 (50 nM)-transfected groups compared to NC (50 nM)-transfected groups. **(C)** Transwell assay (representative micrographs are presented). Si-CREB1 (50 nM) impaired the motility of PC-3 cells. **(D)** Western blot analysis. Si-CREB1 (50 nM) inhibited EMT and AKT/GSK-3β signaling-related protein expression in PC-3 and Du-145 cells. Error bars represent the S.E. obtained from three independent experiments; *P<0.05. Scale bar = 100 μm.

To avoid off-target phenomena, we use three different and effective siRNAs in our studies. The following results were obtained from one of the three siRNAs as a representative. Firstly, CCK-8 assays were performed, and illustrated that CREB1 silencing by Si-CREB1 suppressed PC-3 and Du-145 cell growth at different time points and concentrations (Figure 5A). Secondly, the colony-forming ability was also inhibited in Si-CREB1-transfected group (Figure 5B). We performed Transewell assays to identify the function of CREB1 in prostate cancer cell motility. We found that Si-CREB1 significantly suppressed PC-3 cell migration and invasion ability (Figure 5C).

CREB1 downregulation inhibited AKT/GSK-3β signaling pathway. Si-CREB1-transfected cells exhibited lower level of N-cadherin, Vimentin, phosphorylated AKT (Ser473) and phosphorylated GSK-3β (Ser9), and higher level of E-cadherin (Figure 5D). And CREB1 expression was also included as a control showing the silenced target. We also found that Si-CREB1-transfected cells exhibited decreased expression of c-Met.

### EGFR silencing suppresses prostate cancer cell proliferation, motility and EMT

**Figure 6:**
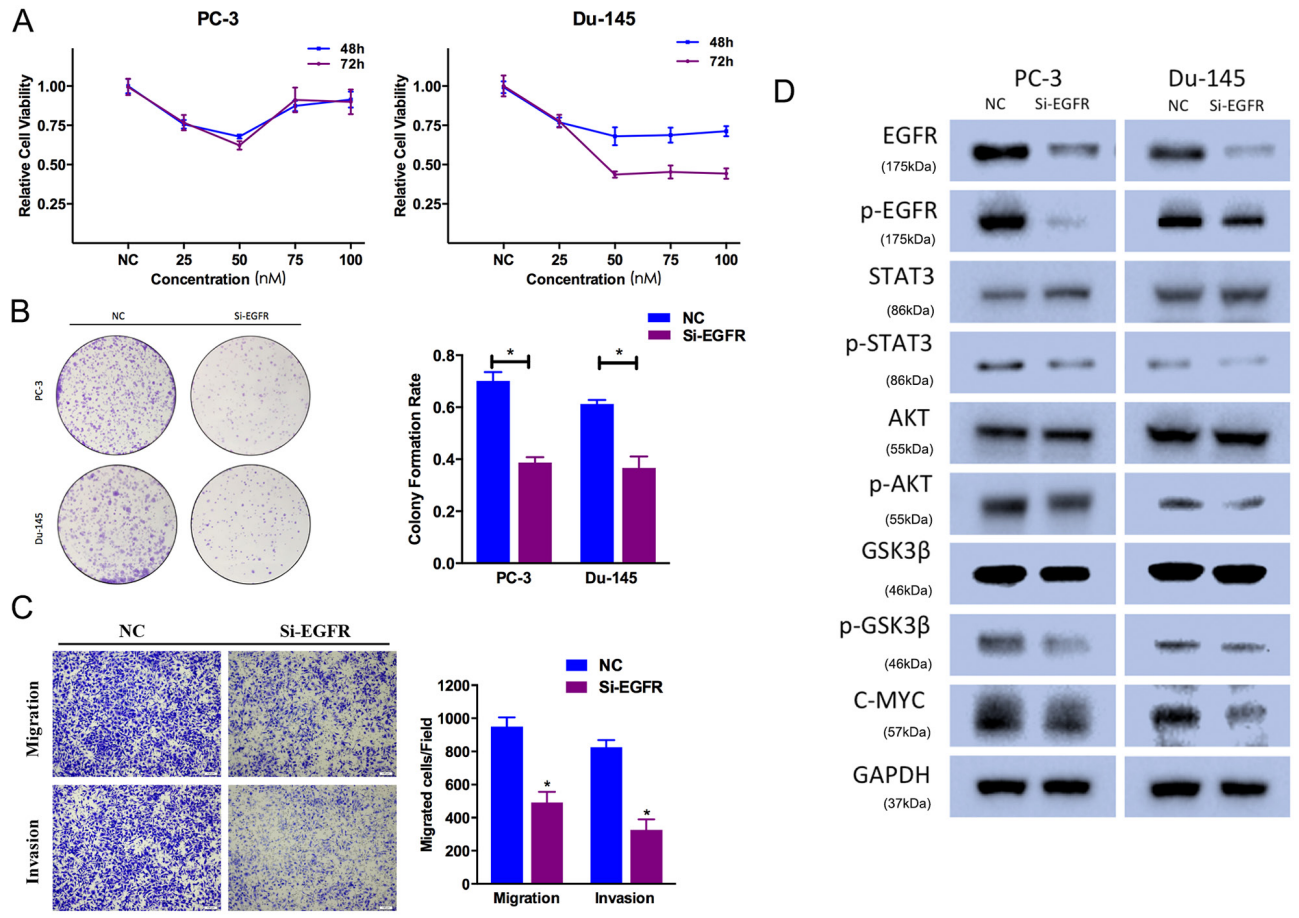
EGFR knockdown suppresses prostate cancer cell proliferation and motility **(A)** CCK-8 assay. The relative cell viability of the Si-EGFR-treated groups of PC-3 and Du-145 cells was lower than that of NC-treated groups (cell viability of 0 nM was regarded as 1.0). **(B)** Colony-formation assay (representative wells are presented). The colony-formation rate was lower for Si-EGFR (50 nM)-transfected groups compared to NC (50 nM)-transfected groups. **(C)** Transwell assay (representative micrographs are presented). Si-EGFR (50 nM) impaired the motility of PC-3 cells. **(D)** Western blot analysis. Si-EGFR (50 nM) inhibited AKT/GSK-3β signaling-related protein expression and p-STAT3 in PC-3 and Du-145 cells. Error bars represent the S.E. obtained from three independent experiments; *P<0.05. Scale bar = 100 μm.

To avoid off-target phenomena, we use three different and effective siRNAs in our studies. The following results were obtained from one of the three siRNAs as a representative. Firstly, CCK-8 assays were performed, and illustrated that EGFR silencing by Si-EGFR suppressed PC-3 and Du-145 cell growth at different time points and concentrations (Figure 6A). Secondly, the colony-forming ability was also inhibited in Si-EGFR-transfected group (Figure 6B). We performed Transewell assays to identify the function of EGFR in prostate cancer cell motility. We found that Si-EGFR significantly suppressed PC-3 cell migration and invasion ability (Figure 6C).

EGFR downregulation inhibited AKT/GSK-3β signaling pathway. Si-EGFR-transfected cells exhibited lower level of phosphorylated AKT (Ser473), phosphorylated EGFR (Tyr1068) and phosphorylated GSK-3β (Ser9) (Figure 6D). And EGFR expression was also included as a control showing the silenced target. Besides, Si-EGFR-transfected cells exhibited decreased expression of phosphorylated STAT3 (Tyr705), which was a key factor in cell proliferation.

**Figure 7:**
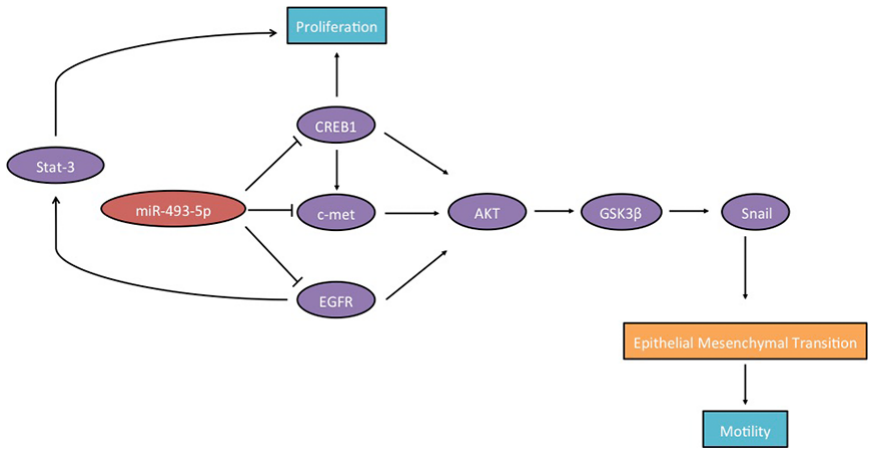
Schematic diagram showing that c-Met, CREB1, EGFR and miR-493-5p establish a regulatory loop in prostate cancer

Figure 7 illustrates the critical outcomes of this study.

## DISCUSSION

Human miR-493-5p belongs to the DLK1-DIO3 imprinted miRNA cluster, and acts as a tumor inhibitor in human cancers [7]. Jia reported that miR-493 mediated DKK1 down-regulation conferred proliferation, invasion and chemo-resistance in gastric cancer cells [10]. Kumar showed that Snail modulated miR-493 formed a negative feedback loop with IGF1R pathway and blocked carcinogenesis [11]. In our study, miR-493-5p expression was lower in prostate cancer blood samples than in benign prostate hyperplasia blood samples. Furthermore, miR-493-5p inhibited the proliferation and motility of prostate cancer cells. These data have indicated that miR-493-5p acts as a tumor suppressor in prostate cancer.

The DLK1-DIO3 region includes the paternally expressed DLK1 and DIO3, as well as the maternally expressed MEG3 and MEG8 [12-14]. Epigenetic changes regulate the DLK1-DIO3 imprinted miRNA cluster. This region contains 53 miRNAs on the forward strand and 1 miRNA on the reverse strand [7]. Previous studies indicated that DLK1-DIO3 miRNA cluster had crucial roles in carcinogenesis. miR-495, miR-409-3p, miR-379 have been found to be related to the pathogenesis and progression of gastric cancer [15]. miR-493 was deregulated in esophageal squamous cell carcinoma [16]. miR-323 was observed to be downregulated in colorectal cancer, and could possibly be a promising biomarker for early diagnosis [17]. miR-433 also locates in the DLK1-MEG3 region, and the CpG islands of miR-433 were hypermethylated. DNA methylation inhibitor, 5-aza-CdR, could increase miR-433 level [9]. In our study, epigenetic programming was involved in decreasing the expression of miR-493-5p in prostate cancer.

Our study investigated the function of miR-493-5p in inhibiting prostate cancer proliferation and motility. We demonstrated novel regulatory circuits involving miR-493-5p, c-Met, CREB1 and EGFR that controlled prostate cancer progression. The above 3 genes were verified as direct downstream targets of miR-493-5p.

c-Met is a well-known receptor tyrosine kinase (RTK), and is frequently overexpressed many human cancers. Recent researches have illustrated that suppression of c-Met could inhibit cancer cell proliferation and invasion, which is a promising method in cancer therapy [18]. In our previous studies, we have confirmed that mir-101, miR-409-3p and miR-433 inhibit bladder cancer cell motility by downregulating c-Met [9, 19, 20]. In our study, miR-493-5p downregulation resulted in the abnormal activation of c-Met in prostate cancer. Furthermore, inhibition of c-Met by miR-493-5p downregulated the activating level of phosphorylated AKT and phosphorylated GSK-3β, resulting in the nuclear localization of Snail, higher level of E-cadherin, and lower level of Vimentin and N-cadherin.

CREB1, as a transcription factor, regulates many downstream genes and implicates in cell proliferation and motility in various cancers. Previous studies indicated that CREB1 also regulated c-Met expression indirectly by increasing the transcription level of MITF, which directly regulated c-Met expression [9]. Additionally, CREB1 involved in regulating EMT via VEGF signaling in prostate cancer [21]. In our study, CREB1 silencing inhibited the AKT/GSK-3β signaling pathway via decreasing c-Met expression.

EGFR family members played important roles in many types of cancers, which resulted in targeted therapies development, including synthesis of small-molecule inhibitors. Nevertheless, activation of the EGFR drove cancer progression via complex mechanisms, which were not well understood [22]. A previous study demonstrated that EGFR promoted prostate tumor-initiating cells and circulating tumor cells survival, and EGFR inhibitor led to suppression of tumor xenograft growth [23].

In conclusion, our study demonstrates that, (1) miR-493-5p is downregulated in prostate cancer possibly due to DNA methylation; (2) miR-493-5p acts as a tumor suppressor in prostate cancer; (3) c-Met, CREB1 and EGFR are miR-493-5p target genes; (4) miR-493-5p suppresses EMT by inhibiting AKT/GSK-3β/Snail signaling pathway in prostate cancer; and (5) CREB1 regulates c-Met/Akt/GSK-3β/Snail signaling pathway indirectly.

Our study identified c-Met, CREB1, EGFR and miR-493-5p establish a regulatory loop in prostate cancer.

## MATERIALS AND METHODS

### Cell lines and cell culture

PC-3 and Du-145 human prostate cancer cell lines, and RWPE-1 human prostate epithelial cell were obtained from the Cell Bank of Type Culture Collection of Chinese Academy of Sciences (Shanghai, China). All cell lines were verified by short tandem repeat (STR) DNA profiling analysis. Cells were cultured as previously reported [6].

### RNA isolation and real-time PCR

The expression level of microRNAs and genes in the cell lines was calculated by quantitative real-time PCR (qRT-PCR). RNA was extracted from cell lines and blood samples (the patient’s whole blood) with RNAiso plus (TaKaRa, Japan) and transcribed into cDNA using PrimeScript RT reagent Kit and One Step PrimeScript miRNA cDNA Synthesis Kit (TaKaRa, Japan). cDNAs were quantified using SYBR Premix Ex Taq (TaKaRa, Japan) with the Bio-Rad iQ5 Real Time PCR System. Small nuclear RNA U6 and GAPDH mRNA were used as internal controls for normalization. All primers sequences are listed in Supplementary Table 1.

### Western blot analysis

Western blot analysis was conducted as previously described [24]. The following primary immunoblotting antibodies were used: anti-GAPDH, anti-E-cadherin, anti-Fibronectin, anti-Vimentin, anti-Snail, and anti-c-Met (Epitomics, Burlingame, CA, USA), anti-β-catenin (Proteintech, Chicago, USA), anti-N-cadherin, anti-c-myc, anti-EGFR, anti-p-EGFR (Tyr1068), anti-GSK3β, anti-p-GSK3β (Ser9), anti-AKT (pan), anti-p-AKT (Ser473), anti-stat3, anti-p-stat3 (Tyr705) and anti-CREB1 (Cell Signaling Technology, Beverly, MA).

### Immunohistochemistry (IHC) staining

IHC staining was performed as previously described [24]. Tumor tissues from mice were analyzed. The strength of positivity was semi-quantified by considering the intensity and the proportion of positive cells.

### Immunofluorescence analysis

Du-145 prostate cancer cells grown on cover slip were fixed in 4% paraformaldehyde (Invitrogen, Cergy-Pontoise, France) for 15 minutes (min). After washed three times with PBS, unspecific sites were blocked using PBS containing 5% BSA at room temperature for 1 h. Cells grown on cover slip were then incubated with anti-E-cadherin (Cell Signaling Technology, Beverly, MA), anti-N-cadherin (Cell Signaling Technology, Beverly, MA), and anti-Vimentin (Epitomics, Burlingame, USA) at 4°C overnight, and incubated with the secondary antibodies Alexa Fluor (Molecular Probes, Life Technologies, Saint-Aubin, France) in the dark at room temperature for 1 h. DAPI were used for Nuclei staining. All stained cells were examined and photographed with a Leica SP5 confocal fluorescence microscope.

### miRNA mimics and small interfering RNA transfections

miRNA-493-5p (miR-493-5p), small interfering RNA-c-Met (Si-c-Met), small interfering RNA-CREB1 (Si-CREB1), small interfering RNA-EGFR (Si-EGFR), and negative control (NC) were purchased from GenePharma (Shanghai, China). Lipofectamine 2000 (Invitrogen, Carlsbad, CA, USA) was used for transfection according to the manufacturer’s instructions. All of the small RNAs were used at a final concentration of 50 nM.

### Cell viability assay

Prostate cancer cells (4000 cells/well) were seeded into 96-well plates. After a 24 h incubation, the cells were treated with miR-493-5p, Si-c-Met, Si-CREB1, or Si-EGFR for 48 h or 72 h incubation. Cell viability assays were performed as previously described [25].

### Colony formation assay

PC-3 and Du-145 prostate cancer cells were seeded into a new six-well plate and incubated for 24 h before treatment with 2′-O-Methyl-modified miR-493-5p, Si-c-Met, Si-CREB1, or Si-EGFR. Detailed experimental procedures were performed as previously described [25].

### Cell migration and invasion assay

A 24-well Boyden chamber with 8 μm pore size polycarbonate membrane (Corning, NY) was used for evaluating cell migration. Matrigel (BD, Franklin Lakes, NJ) was used to pre-coat the membrane to simulate a matrix barrier for evaluating cell invasion. Transwell assays were performed as previously described [25].

### Luciferase reporter assays

The 3′-UTRs (untranslated region) of c-Met, CREB1 and EGFR containing putative miR-493-5p target regions were synthesized (Sangon, Shanghai, China) and cloned between the SacI and SalI sites downstream of the luciferase reporter gene in pmirGLO Dual-Luciferase miRNA Target Expression Vector (Promega, USA). Additionally, the mutant miR-493-5p putative target region was generated using the same approach. All insertions were verified by sequencing (Sangon, Shanghai, China). The dual-Luciferase report assay was performed as previously described [25].

### 5-Aza-2′-deoxycytidine treatment of PC-3 and Du-145 prostate cancer cells

PC-3 and Du-145 prostate cancer cells were treated with 10 μM 5-Aza-2′-deoxycytidine (5-Aza) (Sigma A3656) for 4 days. RNA was extracted and analyzed for miR-493-5p expression.

### DNA methylation analysis

Genomic DNA from PC-3 and Du-145 prostate cancer cell lines was bisulfite modified, and the CpG islands were amplified by PCR (Primers sequences are listed in Supplementary Table 1). The PCR products were separated by agarose gel electrophoresis (3%), extracted and cloned into the pUC18 T-vector (Sangon, China). After bacterial amplification of the cloned PCR fragments by standard procedures, 5 clones were sent for DNA sequencing (Sangon, China).

### Animal experiments

Animal experiments were conducted in accordance with institutional guidelines (1^st^ Affiliated Hospital, College of Medicine, Zhejiang University). Male BALB/c-nude mice (4 weeks old) were purchased from the Shanghai Experimental Animal Center, Chinese Academy of Sciences (Shanghai, China). Lipofectamine 2000-encapsulated miR-493-5p or NC was used for injection. PC-3 cells (1 × 10^8^ in 100 μl PBS) were injected subcutaneously into the right flank of each mouse. When palpable tumors arose, the mice were injected intratumorally with 30μl of Lipofectamine 2000-encapsulated miR-493-5p or NC every 3 days for 15 days. Tumor size was monitored and evaluated every 3 days. Tumor growth was monitored by caliper measurements of the two perpendicular diameters every 3 days, and the volume of the tumor was calculated with the formula V = (width^2^ × length × 0.52).

### Human clinical samples

Prostate cancer blood samples and benign prostate hyperplasia blood samples were obtained from patients diagnosed pathologically. The samples were collected between January 2016 and October 2016 at the First Affiliated Hospital of Zhejiang University, after informed consent of patients and approval of Ethics Committee of 1^st^ Affiliated Hospital, College of Medicine, Zhejiang University (No. 201644).

### Statistical analysis

All statistics were expressed as the mean ± standard error (SE) of three independent experiments. Student’s t-test or two-way ANOVA was used to evaluate the intergroup difference.SPSS for Windows v.21 (SPSS, Chicago, IL) and GraphPad Prism 6.0 (GraphPad Software, La Jolla, CA) were used to conduct the relative analyses. P < 0.05 was considered statistically significant.

## SUPPLEMENTARY MATERIALS FIGURES AND TABLE


