# Supplementary material for: c-Met, CREB1 and EGFR are involved in miR-493-5p inhibition of EMT via AKT/GSK-3β/Snail signaling in prostate cancer
Source: Oncotarget. 2017 Jul 19;8(47):82303–13. doi: 10.18632/oncotarget.19398 (PMC5669891; doi:10.18632/oncotarget.19398)
Supplement: Supplementary file 1 [file oncotarget-08-82303-s001.pdf]

c-Met, CREB1 and EGFR are involved in miR-493-5p inhibition of EMT via AKT/GSK-3β/Snail signaling in prostate cancer

SUPPLEMENTARY MATERIALS

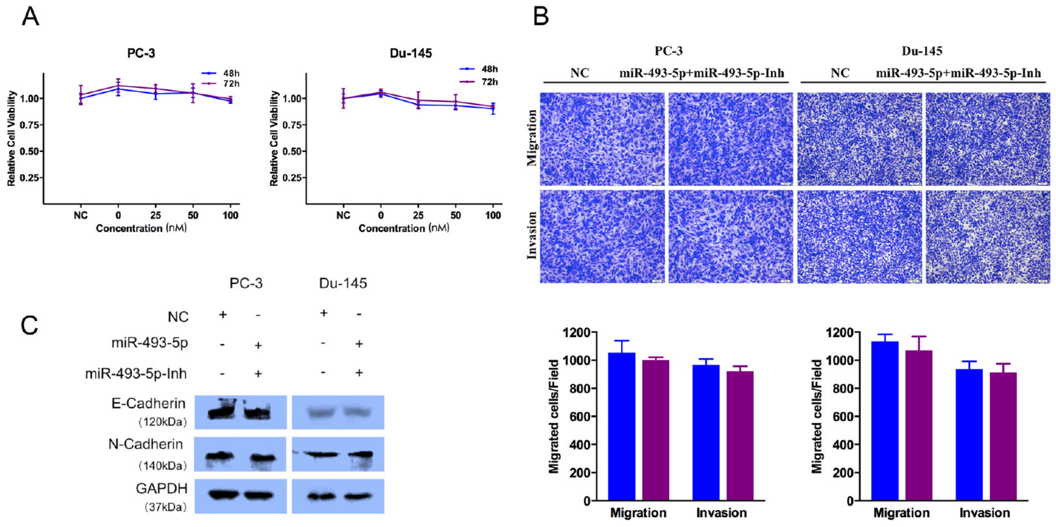

Supplementary Figure 1: Inhibition of miR-493-5p expression partially rescues miR-493-5p-induced suppression of cell proliferation, motility and EMT. (A, B, C) Transfection of miR-493-5p-Inh significantly rescued the miR-493-5p-induced inhibition of cell proliferation, motility and EMT in both prostate cancer cell lines. Error bars represent the S.E. obtained from three independent experiments; Scale bar = 100 μm.

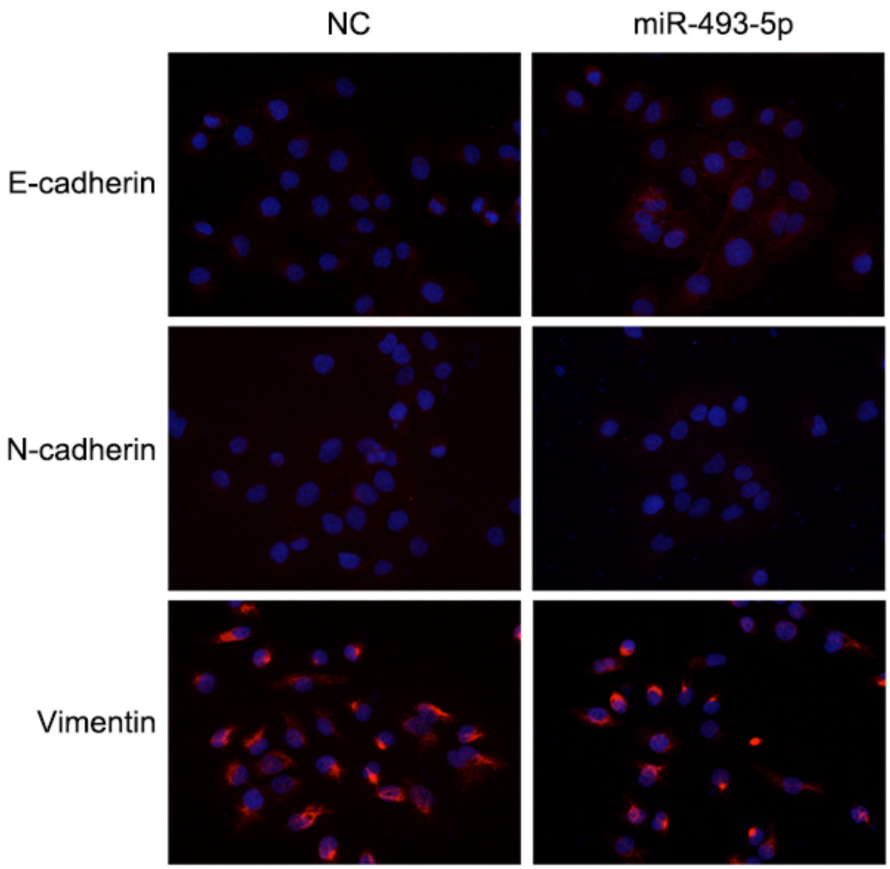

Supplementary Figure 2: Immunofluorescence staining. Immunofluorescence staining illustrates that miR-493-5p overexpression increased the expression of the epithelial marker E-cadherin and decreased the expression of N-cadherin and Vimentin (mesenchymal markers) in Du-145 prostate cancer cell line.

Supplementary Table 1: The oligonucleotides used in this study.

Name[a]	Sequence (5′->3′)
miR-493-5p mimics (sense)	UUGUACAUGGUAGGCUUUCAUU
NC (sense)	ACUACUGAGUGACAGUAGA
miR-493-5p-Inh	AAUGAAAGCCUACCAUGUACAA
NC-Inh	CAGUACUUUUGUGUAGUACAA
Si-c-Met (sense)	GGAGGUGUUUGGAAAGAUAdTdT
	GGAAAGAACCUCUCAACAUdTdT
	GCAACAGCUGAAUCUGCAAdTdT
Si-CREB1 (sense)	GAGAGAGGUCCGUCUAAUGdTdT
	GAGCUGUCCUCUCCAGUAU
	UGAUUCAAACAGUAGUCAA
Si-EGFR (sense)	GCAGAUCAUCAGAGGAAAUdTdT
	GCAACAUGUCGAUGGACUUdTdT<br>GGAGAUAAGUGAUGGAGAUdTdT
miR-493-5p F	TTGTACATGGTAGGCTTTCATT
U6 F	TGCGGGTGCTCGCTTCGGCAGC
Pre-miR-493-5p F	tcgaGCTAGCCTCCGAAGCAAACAATGAAA
Pre-miR-493-5p R	tcgaAAGCTTCGTCTACAAGGACTAACCGAAA
Methylation PCR F	TTATATTTGGGAATTAGTTATGTYGTT
Methylation PCR R	CCCAAATTCTATAACAAATTACTCTAAC
c-Met F	TGTCCCGAGAATGGTCATAA
c-Met R	AGGGAAGGAGTGGTACAACA
CREB1 F	ATTCACAGGAGTCAGTGGATAGT
CREB1 R	CACCGTTACAGTGGTGATGG
EGFR F	AACACCCTGGTCTGGAAGTACG
EGFR R	TCGTTGGACAGCCTTCAAGACC
GAPDH F	AAGGTGAAGGTCGGAGTCA
GAPDH R	GGAAGATGGTGATGGGATTT
c-Met-Wt F	cTGTTTATAAATGAACAGGATGTAATGTACATAGATGACATTAAGAAAg
c-Met-Wt R	tcgacTTTCTTAATGTCATCTATGTACATTACATCCTGTTCATTTATAAACAgagct
c-Met-Mut F	cTGTTTATAAATGAACAGGATATGTAATGCATAGATGACATTAAGAAAg
c-Met-Mut R	tcgacTTTCTTAATGTCATCTATGCATTACATATCCTGTTCATTTATAAACAgagct
CREB1-Wt F	cAATGCATTTTATTAACACTATGTACATAATAGCTGCTTTGTGTTCAg
CREB1-Wt R	tcgacTGAACACAAAGCAGCTATTATGTACATAGTGTTAATAAAATGCATTgagct
CREB1-Mut F	cAATGCATTTTATTAACACTtacatgtTAATAGCTGCTTTGTGTTCAg
CREB1-Mut R	tcgacTGAACACAAAGCAGCTATTAACATGTAAGTGTTAATAAAATGCATTgagct
EGFR-Wt F	cGCAATGATGAAAGCAGTATTTGTACAAATGAAAAGCAGAATTCTCTTg
EGFR-Wt R	tcgacAAGAGAATTCTGCTTTTCATTTGTACAAATACTGCTTTCATCATTGCgagct
EGFR-Mut F	cGCAATGATGAAAGCAGTATTacatgttATGAAAAGCAGAATTCTCTTg
EGFR-Mut R	tcgacAAGAGAATTCTGCTTTTCATAACATGTAATACTGCTTTCATCATTGCgagct

[a] F, forward primer; R, reverse primer.
